# YOLOv5 with Channel Attention and Feature Fusion for Wheel Surface Defect Detection

**DOI:** 10.3390/s26082410

**Published:** 2026-04-15

**Authors:** Juanjuan Gao, Yuanchao Wang, Xiuye Xia, Zhenyu Zhang, Fucai Liu

**Affiliations:** 1 Engineering Research Center of the Ministry of Education for Intelligent Control System and Intelligent Equipment, Yanshan University, Qinhuangdao 066004, China; 2 Key Laboratory of Industrial Computer Control Engineering of Hebei Province, Yanshan University, Qinhuangdao 066004, China

**Keywords:** wheel surface defect detection, deep learning, YOLO, object detection, machine vision

## Abstract

Wheels play an important role in automobiles. However, wheel surface defects may have an impact on the safety of the vehicle. Therefore, there is a need to identify the wheel defects. Unlike other common defects, there is a wide variety of wheel surface defects, and the wheel surface contains curved surfaces, which present a challenge for detection. In response to these problems, this paper proposes a wheel surface defect detection algorithm based on an improved YOLOv5. The improved algorithm embeds the efficient channel attention mechanism (ECA) and the adaptive spatial feature fusion (ASFF) detection head into the network and uses the structure of GSConv+SlimNeck. The improvement enables the algorithm to select the best convolutional kernel during feature extraction and fully fuse the features of different scales, enhancing the detection effect of small objects. The experimental results show that the improved YOLOv5 algorithm achieves higher mAP and detection accuracy while maintaining a high FPS, which meets the requirements for real-time detection.

## 1. Introduction

The wheel is one of the crucial components of the automobile. In the production process, automotive wheels may have surface defects for various reasons, which will affect the aesthetics and performance of the wheel [1]. Therefore, wheel surface defect detection is extremely significant. At present, wheel surface defect detection mainly relies on manual inspection. Workers are required to manually rotate the wheel in front of the workstation to observe different surfaces. It takes about 20–30 s to complete the inspection of a hub. Repetitive and intense labor over a long period can affect the workers’ inspection accuracy. Workers with different experience levels may have different judgments on similar defects. Additionally, there exists a scarcity of research on wheel surface defect detection. Therefore, it is necessary to invent an automatic method for detecting defects on the surface of the wheel.

Currently, other defect detection algorithms are mainly object detection methods based on image processing techniques. Defect detection algorithms are mainly divided into traditional machine vision algorithms and deep learning algorithms. Traditional defect detection methods are typically based on handcrafted visual features and machine learning classifiers, such as support vector machine (SVM) [2], random forest [3], etc. However, due to the difficulty of acquiring defects on the surface of the wheel hub and the difficulty of defect extraction, it is difficult for traditional machine vision algorithms to achieve a high detection accuracy.

**Figure 1:**
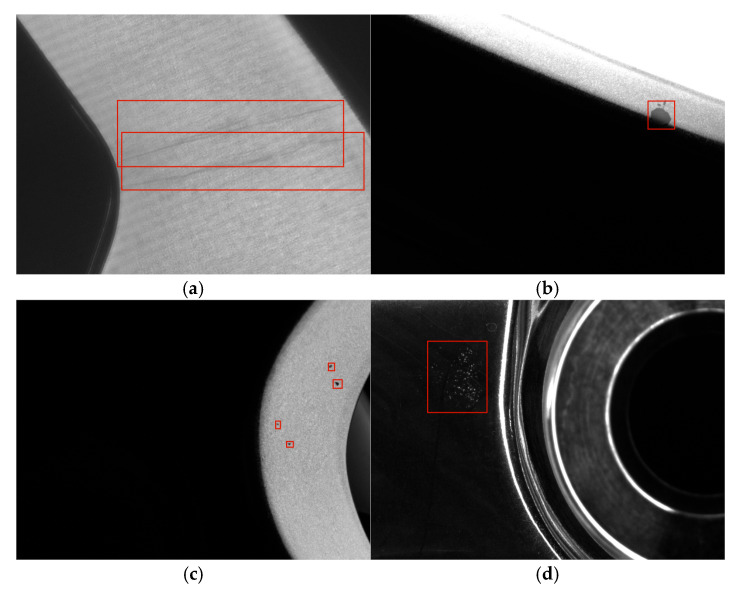
Different kinds of wheel surface defects. Defects have been marked in the pictures. (**a**) Linear, (**b**) sludge, (**c**) dotted, (**d**) pinhole.

Most surface defects have a wide range of categories. Among the various defects, there are four defects: linear defects, sludge defects, dotted defects and pinhole defects are studied in this paper and are shown in Figure 1. Unlike traditional surface defects (such as steel surface defects), the difficulties that exist in wheel surface defect detection are as follows: The wheel surface is curved rather than flat, and the background of wheel surface defects is more complex than common defects. Moreover, the hollowed-out parts under different shooting angles may cause interference. Among the four defects mentioned above, the area of dotted defects is relatively small and belongs to small targets. Pinhole defects appear in clusters and are not fixed in shape or size. Based on the analysis above, this paper presents a YOLOv5-based algorithm for wheel surface defect detection. The main contributions of this paper are shown below:

(1) The C3 module with Efficient Channel Attention (C3 ECA) module was introduced in the backbone part of YOLOv5. The ECA module was added to the back of the first conv module in the C3 module, which improves the feature extraction ability of the network.
(2) In the neck part of YOLOv5, the Feature Pyramid Network+Path Aggregation Network (FPN+PAN) structure is replaced by the SlimNeck structure and the lightweight GSConv module is used to reduce the computational cost and complexity of the network.
(3) In the head part of YOLOv5, the detection head is replaced with the Adaptive Spatial Feature Fusion (ASFF) detection head, which can fully fuse three different sizes of feature maps and improve the effectiveness of small object detection.

The rest of this paper is organized as follows. Section 2 introduces the deep learning object detection algorithms, the attention mechanism and the YOLOv5 algorithm. Section 3 details the proposed method. Section 4 lists the experimental results and analyses, and Section 5 is the conclusion.

## 2. Related Work

### 2.1. Deep Learning Object Detection

Object detection algorithms are mainly divided into two categories: one-stage object detection algorithms and two-stage object detection algorithms. One-stage object detection algorithms mainly include Single Shot MultiBox Detector (SSD) [4], the YOLO [5,6,7,8] series of algorithms, and so on. Two-stage object detection algorithms mainly include Region-based Convolutional Neural Network (R-CNN) [9], Fast R-CNN [10], Faster R-CNN [11], and so on. Sun et al. [1] proposed a wheel surface defect detection method based on an improved Faster R-CNN. The authors used the faster ZFNet and also improved the Region Proposal Network (RPN) network. The improved ZFNet was used as a pre-training network and achieved 72.9% mAP. Ding et al. [12] replaced the VGG16 network architecture in the original SSD algorithm with the DenseNet network architecture and used a migration learning approach. The detection accuracy of three kinds of wood surface defects reached 96.1%, which was about 5% higher than the original algorithm. Li et al. [13] implemented a defect detection algorithm based on the improved Cascade R-CNN algorithm. The improved algorithm adds a feature pyramid network (FPN) to the original algorithm, and the detection accuracy was improved by 4.09%. Fan et al. [14] proposed a real-time apple defect detection method based on YOLOv4. In order to achieve better real-time performance, the authors used channel pruning and layer pruning and proposed a non-maximal value suppression method based on the L1 criterion. The inference speed of the improved algorithm was reduced by 10.82 ms and the mAP reached 93.74%. Guo et al. [15] proposed an MSFT-YOLO model based on CNN and Transformer. The authors introduced the TRANS module in the backbone and neck parts of the YOLOv5l model and used a bidirectional feature pyramid network in the neck part. MSFT- YOLO improves the mAP by about 7% over the original algorithm. Cheng et al. [16] proposed a new deep convolutional network called DEA RetinaNet. The network first embedded a new channel attention mechanism in the network and secondly used adaptive spatial feature fusion to achieve 78.25% mAP on the NEU-DET dataset. Liu et al. [17] proposed a fast power line edge detection method. In order to detect the critical parts accurately, the authors introduced RepVGG, a diverse branch block (DBB), and an ECA attention mechanism to obtain RepYOLO. Moreover, using an embedded platform for optimization and acceleration increased the inference speed by a factor of four. The proposed method improves accuracy by 1.2% over the original algorithm.

### 2.2. Attention Mechanism

Introducing attentional mechanisms into neural networks can improve processing efficiency and accuracy by making it easier for the network to pay attention to critical information and reduce the level of attention paid to other useless information. Hu et al. [18] proposed the Squeeze-and-Excitation (SE) Attention Module, which focuses on the channel relationships and can be easily added to mainstream neural networks. Woo et al. [19] proposed a convolutional block attention module (CBAM), which employs two independent attention mechanisms, channel attention and spatial attention, and is capable of adaptive feature refinement. Wang et al. [20] proposed the efficient channel attention (ECA) module, which replaces the Multilayer Perceptron (MLP) module in SEBlock with a one-dimensional convolution, effectively reducing the number of parameters.

### 2.3. YOLOv5

The YOLO algorithm was initially introduced by J. Redmon et al. [5] in 2015. The algorithm treats the object detection problem as a regression problem by taking the whole image directly as input to the network and dividing it into S × S grids. When the center of an object falls within a grid, that grid is responsible for predicting the object, yielding B bounding boxes and confidence levels. These confidence levels reflect the accuracy of the identified object and the precision of the bounding boxes, respectively.

**Figure 2:**
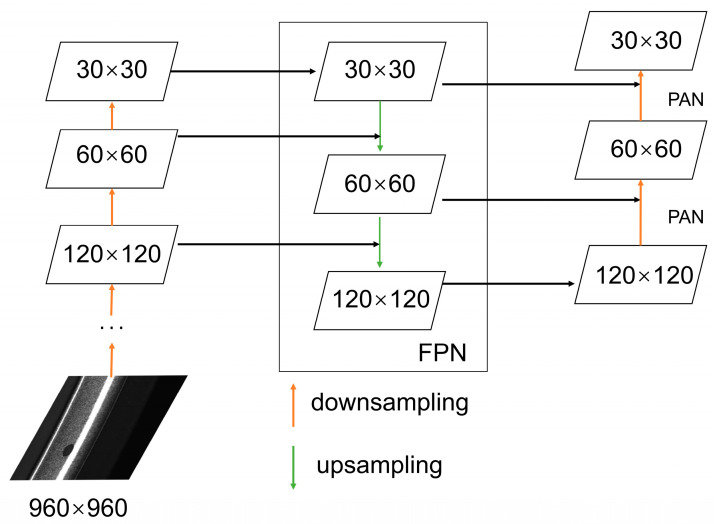
Feature Pyramid Network+Path Aggregation Network (FPN+PAN) structure diagram.

The YOLOv5 algorithm is the fifth version of the YOLO series of algorithms, which has more advantages in speed and accuracy. There are four versions of the YOLOv5 algorithm: YOLOv5s, YOLOv5m, YOLOv5l, and YOLOv5x. The four versions differ only in the width and depth parameters of the network. Among them, YOLOv5s has the fastest detection speed but is not as good as the other models in terms of detection accuracy. The structure of the YOLOv5 algorithm can be mainly divided into three parts: backbone, neck and head. The backbone part uses the structure of CSPDarknet53, which contains several residual modules. The backbone network mainly performs a series of convolutional operations on the input image and obtains feature maps of different sizes through downsampling. The neck part uses the FPN+PAN structure, which mainly fuses the feature maps of each layer. The FPN conveys the semantic features from the top down, and the PAN conveys the positional information from the bottom up. The head part is capable of outputting three feature maps of different sizes, corresponding to different scales of the prediction boxes, containing information about the location, size, and category of the target defects. The FPN+PAN structure is shown in Figure 2.

## 3. Proposed Method

**Figure 3:**
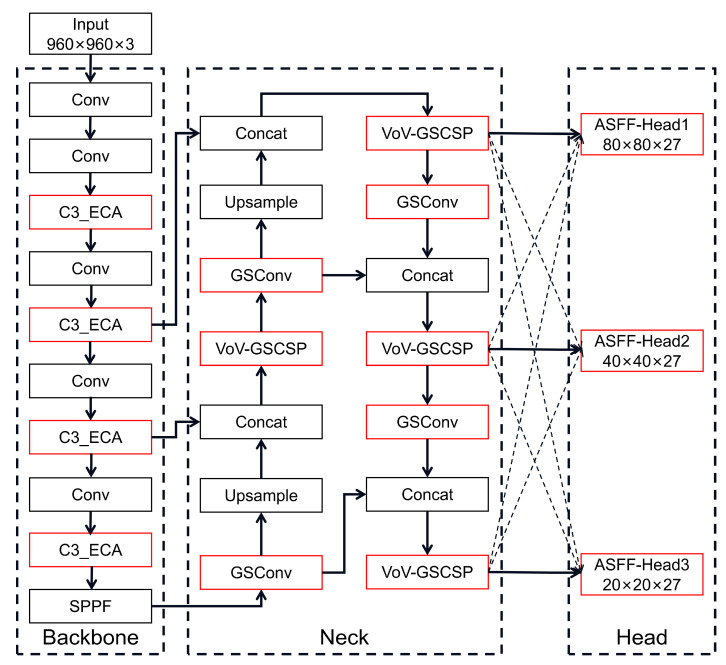
The improved YOLOv5s network structure.

In this section, we focus on the improved YOLOv5s structure. As a representative method of one-stage detectors, the YOLO algorithm has high detection accuracy and detection speed. Our network mainly improves the YOLOv5s model, and the structure of the improved YOLOv5s model is shown in Figure 3. Firstly, the ECA module is inserted into the C3 module in the backbone network to improve the feature extraction capability, then the GSConv+SlimNeck structure is used to reduce the complexity of the model, and finally, the detection head is replaced with the ASFF Detect head to improve the small object detection effect.

As shown in Figure 3, the proposed model follows the original YOLOv5s framework and introduces three modifications in the backbone, neck, and head, respectively. In the backbone, the ECA module is embedded into the C3 blocks to form the C3-ECA module for enhancing channel interaction during feature extraction. In the neck, the original convolution layers are replaced with GSConv, and the original C3 modules are replaced with VoV-GSCSP to construct the SlimNeck structure. In the head, the original YOLOv5s detection head is replaced by a three-scale ASFF-Detect head, which performs adaptive fusion on feature maps of different resolutions before prediction.

### 3.1. ECA Net

**Figure 4:**
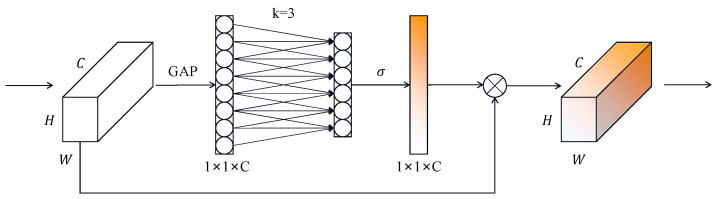
Efficient Channel Attention (ECA) structure diagram.

Due to the complexity of the wheel surface defect background, it is easy to interfere with the extraction of defect features. Therefore, we introduce the ECA [20] attention mechanism into the backbone of the YOLOv5 network and insert the ECA attention mechanism into the first convolutional module in the backbone of the C3 module, which is able to learn the correlation between the channels and adaptively adjust the weights of the channels to improve the detection accuracy without significantly increasing the number of parameters in the model. The ECA attention mechanism is mainly improved from the SE [18] attention mechanism. The SE attention mechanism mainly consists of two steps: compression and excitation. In the squeeze step, the input feature map is compressed into a vector by a global average pooling operation and then mapped to a smaller vector by a fully connected layer. In the excitation step, each element of the vector is used as an input to the sigmoid activation function, and the resulting output is then multiplied by the original input feature map to obtain a weighted feature map. Although it improves the performance of the network, it increases the computational complexity. ECA reduces the computational complexity by replacing the MLP (FC+ReLU+FC+Sigmoid) in SE with a one-dimensional convolution. The structure of the ECA module is shown in Figure 4.

In the ECA module, the original feature map of size H × W × C is first compressed into a 1 × 1 × C feature descriptor by global average pooling. Then, a one-dimensional convolution is applied to model local cross-channel interaction and generate the channel attention weights. Different from SE, ECA avoids dimensionality reduction and determines the convolution kernel size adaptively according to the channel dimension C. Following the original ECA design, the adaptive kernel size can be expressed as

(1) $$k=\psi \left(C\right)={/\frac{\log_{2}C+b}{a}/}_{\mathrm{odd}},$$

where γ and b are constants, and $|\cdot {|}_{odd}$ denotes the nearest odd integer. In this paper, a=2 and b=1. In this way, the ECA module can adaptively select an appropriate one-dimensional convolution kernel size according to the channel dimension, thereby achieving efficient local cross-channel interaction with low computational overhead.

**Figure 5:**
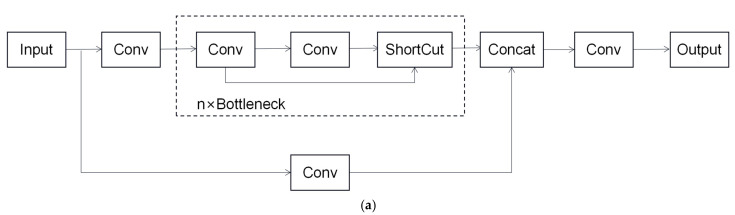

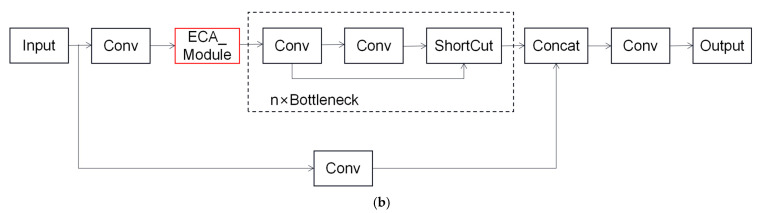
C3 (**a**) and C3 ECA (**b**) structure diagram.

In order to enhance the feature extraction capability of the C3 module in the backbone, we insert the ECA attention mechanism module into the C3 module. The structure of the C3 module and the C3 ECA module is shown in Figure 5.

### 3.2. GSConv+SlimNeck

In order to reduce the complexity of the model while maintaining the detection accuracy as much as possible, we use the structure of GSConv+SlimNeck [21] in the neck part. Firstly, the ordinary convolutional module in the neck part is replaced by the lightweight GSConv module, and then the C3 module in the neck part is replaced by the VoV-GSCSP module.

**Figure 6:**
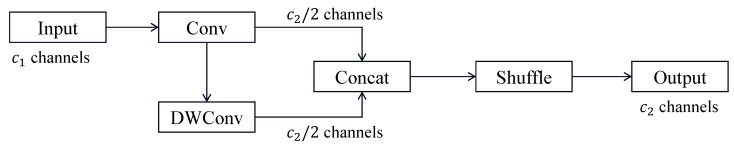
GSConv structure diagram.

The structure of GSConv is shown in Figure 6. GSConv uses a new method, shuffle, which allows the information from the standard convolution to be fully blended into the output of the depth-separable convolution. The method allows the output of GSConv to be as close as possible to the output of a standard convolution while reducing computational cost.

**Figure 7:**
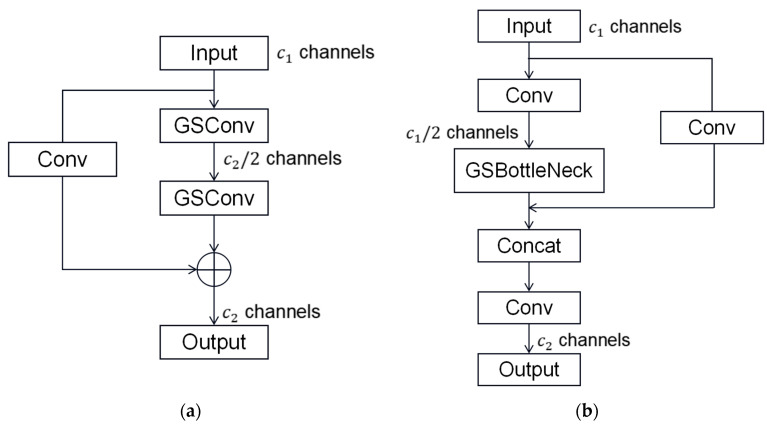
GSBottleneck (**a**) and VoV-GSCSP (**b**) structure diagram.

Based on GSConv, GSBottleneck is introduced, and the structure is shown in Figure 7a. Then, the one-time aggregation method is used to obtain the cross-stage partial network module VoV-GSCSP, which reduces the complexity of the network. The structure diagram is shown in Figure 7b.

The backbone part of the network mainly performs downsampling operations on the input image, and the semantic information will be lost each time the image is compressed or the number of channels is expanded. GSConv retains most of the hidden connections between the channels, which is better for processing the feature maps. Since the feature maps are no longer compressed in the neck part of the network, using the GSConv+SlimNeck structure in this part can process less information and give better results.

### 3.3. ASFF-Detect

**Figure 8:**
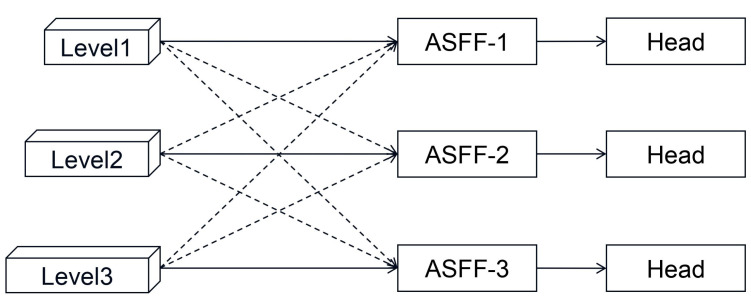
Adaptive Spatial Feature Fusion Detect (ASFF-Detect) structure diagram.

In the original YOLOv5 algorithm, the feature fusion approach of FPN+PAN is used to fuse the high-level semantic information and the low-level location information. However, this structure only uses articulation or summation, which only partially utilizes the features at different scales. Liu et al. [22] proposed a new feature fusion approach, ASFF, to replace the above fusion approach. The structure of ASFF is shown in Figure 8.

In Figure 8, level 1, level 2 and level 3 are three feature maps with sizes of 80 × 80, 40 × 40 and 20 × 20, respectively. We take ASFF-2 as an example. For level 1, the number of channels in the feature map needs to be adjusted to be the same as level 2 and adjusted to the same size after downsampling. Similarly, for level 3, the number of channels of the feature map needs to be adjusted to be the same as level 2 and adjusted to the same size after upsampling. The features on each layer are first multiplied by the weight parameters and then the result of the multiplication is summed to obtain the fused features on the second layer. The process can be shown by the formula below:

(2) $${y^{l}}_{ij}={\alpha^{l}}_{ij}\cdot {{x^{1}}_{ij}}^{\to l}+{\beta^{l}}_{ij}\cdot {{x^{2}}_{ij}}^{\to l}+{\gamma^{l}}_{ij}\cdot {{x^{3}}_{ij}}^{\to l}$$

where ${y^{l}}_{ij}$ denotes the (i, j)th feature vector of the output; ${\alpha^{l}}_{ij}$, ${\beta^{l}}_{ij}$, and ${\gamma^{l}}_{ij}$ are the weight parameters of the three feature maps; and ${{x^{1}}_{ij}}^{\to l}$, ${{x^{2}}_{ij}}^{\to l}$, and ${{x^{3}}_{ij}}^{\to l}$ are the (i, j)th feature vectors of the first, second, and third layers transformed to the lth layer, respectively. Moreover, the weight parameters satisfy the following conditions: ${\alpha^{l}}_{ij}$ + ${\beta^{l}}_{ij}$ + ${\gamma^{l}}_{ij}$ = 1, ${\alpha^{l}}_{ij}$, ${\beta^{l}}_{ij}$, ${\gamma^{l}}_{ij}$∈ [0, 1].

## 4. Experiments and Results

### 4.1. Datasets and Preprocessing

The wheel surface defect dataset employed in this paper is a self-constructed dataset. The defect pictures were captured mainly from the front and side surfaces of the wheel using a Basler acA3800-10gm industrial camera (Basler AG, Ahrensburg, Germany). During image acquisition, a CST light (Dongguan CST Automation Technology Co., Ltd., Dongguan, China) source was used to provide stable illumination conditions. The dataset focuses on four different types of defects: linear defects, sludge defects, dotted defects, and pinhole defects. Each kind of defect comprises 120 pictures, totaling 480 defect pictures. The original resolution of the images is 960 × 704. Considering the influence of the dataset size on the training results, the original dataset was first divided into the training set, validation set, and test set according to a ratio of 7:2:1, containing 336, 96, and 48 defect images, respectively. Then, only the training set was augmented by rotating 90°, 180°, and 270°, and by horizontal and vertical flipping, so that the number of training images was expanded to 2016. The validation set and test set were not augmented and were used only for model validation and performance evaluation.

### 4.2. Experimental Environment and Implementation Details

The experiments use a Sugon remote computing platform (Dawning Information Industry Co., Ltd. (Sugon), Beijing, China) with a 7285-32C-2.0GHz processor, 128 G RAM, 200 Gb computing network and four heterogeneous acceleration cards. Each experiment occupies four nodes, 32 CPUs and four DCU accelerator cards (Hygon Information Technology Co., Ltd., Beijing, China). The experiments use the PyTorch (v2.1.0) deep learning framework and Python (v3.6). In the training phase, the stochastic gradient descent (SGD) optimizer is used, with weight decay set to 0.0005, momentum set to 0.937, initial learning rate set to 0.01, input image size set to 960 × 960, batch size set to 32, and epochs set to 100.

In addition, official pre-trained weights were used for transfer learning during training. The data augmentation strategy included image rotation, horizontal flipping, vertical flipping, and Mosaic and MixUp augmentation, where the probabilities of Mosaic and MixUp were set to 1.0 and 0.25, respectively. These settings were used to improve the robustness and generalization ability of the model.

All compared models, including Faster R-CNN, SSD, YOLOv4, and YOLOv5s, were trained and evaluated on the same dataset split under the same hardware/software environment and consistent major training settings. The input image size was 960 × 960, the batch size was 32, the initial learning rate was 0.01, and the number of epochs was 100.

### 4.3. Evaluation Metrics

In object detection, the evaluation metrics used in this paper include precision, recall, average precision (AP), mean average precision (mAP), and frames per second (FPS). Precision represents the proportion of positive predictions that are correct, whereas recall represents the proportion of actual positive samples that are correctly detected. Since precision and recall alone are insufficient to comprehensively evaluate the detection performance, AP and mAP are employed as the primary metrics. AP is defined as the area under the precision–recall (PR) curve, and mAP is the average AP over all defect categories. In this study, AP/mAP is reported at an IoU threshold of 0.5, corresponding to mAP@0.5. In addition, during inference, the confidence threshold is set to 0.25, the NMS IoU threshold is set to 0.45, and the input image resolution is 960 × 960. The formulas for these evaluation metrics follow the standard definitions commonly used in object detection evaluation and are shown below [23]:

(3) $$\mathrm{Precision}=\frac{\mathrm{TP}}{\mathrm{TP}+\mathrm{FP}}\;\times \;100\%,$$

(4) $$\mathrm{Recall}=\frac{\mathrm{TP}}{\mathrm{TP}+\mathrm{FN}}\;\times \;100\%,$$

where TP is the defect predicted by the model as a defect, FP is the background predicted by the model as a defect, FN is the defect predicted by the model as a background, and TN is the background predicted by the model as a background.

(5) $$AP=\int_{0}^{1}p(r)dr\;$$

(6) $$\mathrm{mAP}=\frac{\sum_{i=1}^{K}{\mathrm{AP}}_{i}}{K},$$

where p(r) represents the precision at recall r. Therefore, AP can be interpreted as the area under the precision–recall (PR) curve. K is the number of categories. When multiple categories exist in the dataset, mAP is the average of the AP of each category.

### 4.4. Experimental Results and Analysis

Experimental Results: Table 1 shows the training results of various common object detection algorithms on the wheel surface defect dataset. In future industrial inspection scenarios, the detection speed and detection accuracy of the algorithms are important factors to measure the performance of the algorithms. Therefore, we compared the mAP and FPS of each algorithm in this section.

As can be seen from Table 1, the SSD algorithm has the highest average precision on dotted defects, the original YOLOv5 algorithm has the fastest detection speed, and the improved YOLOv5 algorithm improves the AP and mAP on almost all of the four defects. Although SSD and YOLOv4 also have high accuracy on linear defects, point defects, and sludge defects, both perform poorly on pinhole defects, and thus, the mAP is slightly worse than that of YOLOv5s. The improved YOLOv5 algorithm proposed in this paper has the best performance with stronger feature extraction and feature fusion capabilities and can better handle complex background information.

Among the four types of defects, sludge defects have a larger area and are easier for feature extraction. The ground-truth boxes of linear defects have a large aspect ratio, and the shape of dotted defects is a fixed origin. All the above three defects have more obvious features, so the AP is higher. Pinhole defects have a lower AP because they often occur in clusters and do not have a fixed shape or boundary. Adding more datasets can be a straightforward way to improve the AP of pinhole defects. Although the proposed model reduces the inference speed from 77 FPS to 48 FPS compared with the original YOLOv5s, the speed is still sufficient for real-time industrial inspection in most wheel surface defect detection scenarios. In practical applications, detection accuracy is often more critical than pursuing the maximum inference speed, since missed detections and false detections may directly affect product quality control. Therefore, the proposed method can be regarded as a trade-off between accuracy and speed: it sacrifices part of the inference efficiency to obtain better feature extraction and multi-scale fusion capability, thereby improving the overall detection performance, especially for complex backgrounds and small defects.

### 4.5. Ablation Study

In order to verify the effectiveness of each improvement, ablation experiments were conducted on each improved model. As can be seen from Table 2, there is a gradual increase in mAP after each improvement, and the improved algorithm proposed in this paper improves on mAP by 2.7% over the original YOLOv5s algorithm. Giga Floating-point Operations Per Second (GFLOPs) are used to measure the computational complexity of the model. In general, higher GFLOPs indicate a larger amount of computation and higher inference cost, rather than better GPU performance. Params (M) is the number of parameters of the model and is measured in millions. It can be seen that the algorithm with improved head and neck parts reduces the GFLOPs while having the lowest number of parameters due to the use of lightweight modules. The algorithm proposed in this paper has the highest GFLOPs but increases the number of parameters more due to the incorporation of the ASFF-Detect detection header, which adds some operations in the fusion stage of the multilayer feature map. Although FPS decreases slightly, it can still meet the requirements of real-time detection in industry.

### 4.6. Detection Results and Analysis

**Figure 9:**
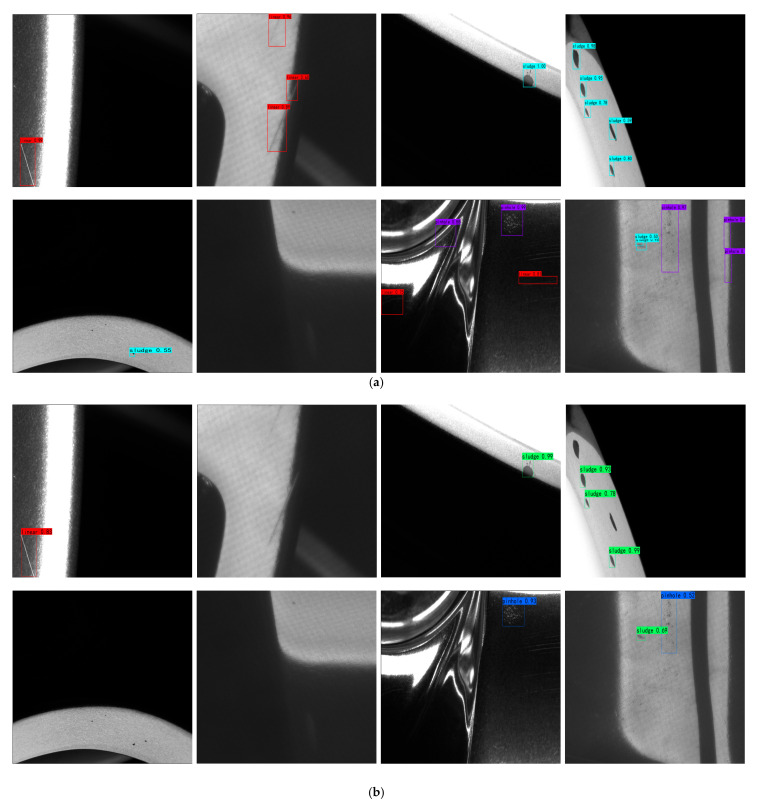

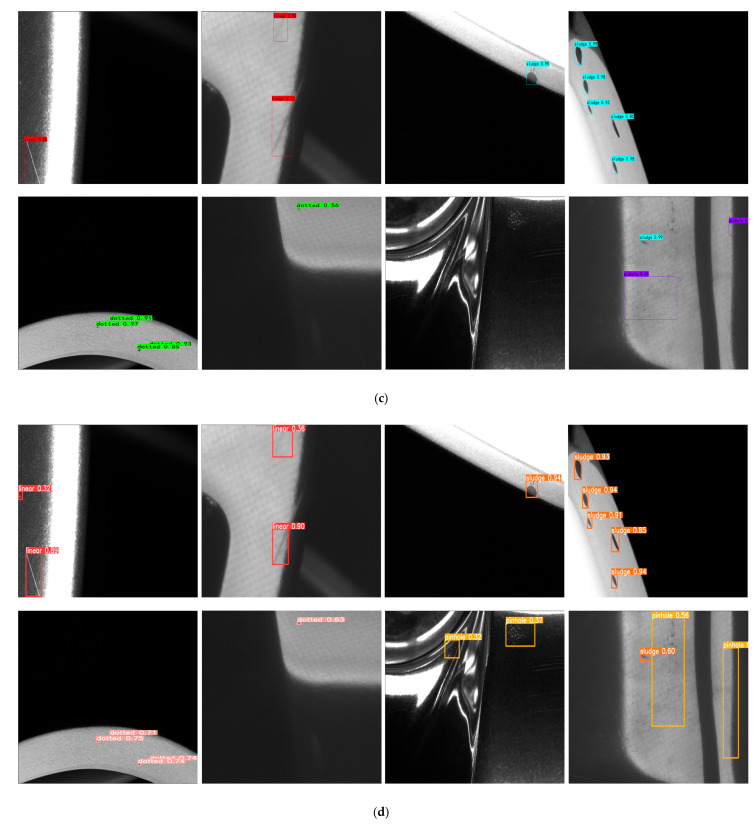

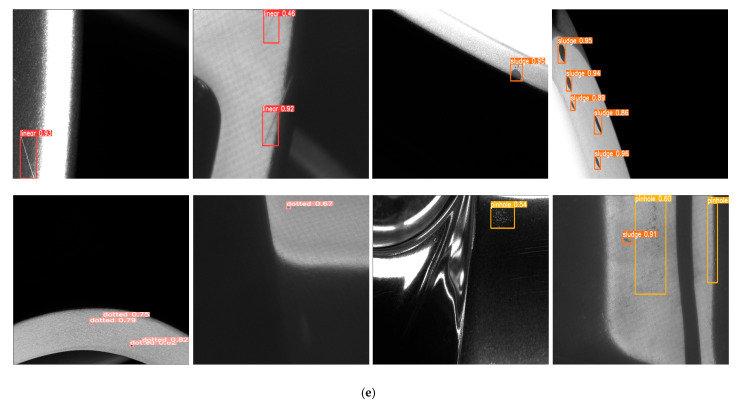
Some visual detection results on the wheel surface defect dataset. (**a**) Some detection results of Faster R-CNN. (**b**) Some detection results of SSD. (**c**) Some detection results of YOLOv4. (**d**) Some detection results of YOLOv5s. (**e**) Some detection results of our method.

To provide a more comprehensive evaluation of the proposed method, additional error analysis was conducted from the perspectives of category-wise performance, small-object detection, and typical failure cases. Figure 9 gives some of the detection results of each algorithm in Section 4, featuring two examples for each type of defect and eight in total. From the figure, it can be seen that among the four kinds of defects, the sludge defects have the best detection effect, while the pinhole defects have the worst detection effect. The Faster R-CNN algorithm and SSD algorithm have a more serious situation of leakage and misdetection. Compared with the original algorithm, the improved YOLOv5s algorithm improves the confidence level, and the misdetection of pinhole defects has been solved to some extent. However, the inaccurate detection of linear defects has not been solved, which proves that the algorithm still has room for improvement.

## 5. Conclusions

In this paper, we propose a YOLOv5-based wheel surface defect detection algorithm. For the complex background of the wheel surface, we insert the ECA attention mechanism into the C3 module of the backbone network to filter out irrelevant background information while extracting features. In order to reduce the complexity of the model while maintaining the original accuracy, we use the structure of GSConv+SlimNeck in the neck part, which better balances the accuracy and speed of the model. To better utilize the semantic and positional information in feature maps of different sizes, we replaced the detection head with the ASFF-Detect head to improve the detection of small targets. On the wheel surface defect detection dataset, the improved YOLOv5s algorithm achieves 75.3% mAP, which is 2.7% higher than the original YOLOv5s algorithm, and the FPS of 48 can also meet the requirements for real-time industrial inspection. Therefore, the proposed method provides a practical trade-off between detection accuracy and inference speed for industrial wheel surface defect inspection. However, this study still has several limitations. First, the dataset size is still relatively limited, which may restrict the generalization ability of the model in more complex industrial environments. Second, the proposed method still shows relatively limited detection performance on pinhole defects, indicating that this type of defect remains challenging due to its clustered distribution, irregular shape, and unclear boundaries. Third, although the proposed method improves the overall detection accuracy, the incorporation of the ASFF-Detect head increases the model complexity and reduces the inference speed compared with the original YOLOv5s. In future research, we will consider using more improvements, such as improving the loss function of the algorithm and increasing the number of detection heads. We will also expand the dataset, especially the number and diversity of pinhole defect samples, or use more data augmentation methods to enhance the model’s generalization and detection accuracy.

## Figures and Tables

**Table 1:** Accuracy and speed comparison of different algorithms on the same wheel surface defect data.

Method	Linear	Dotted	Sludge	Pinhole	mAP	FPS
Faster R-CNN	0.677	0.102	0.607	0.365	0.438	28
SSD	0.662	0.820	0.753	0.308	0.636	30
YOLOv4	0.671	0.776	0.782	0.313	0.636	28
YOLOv5s	0.704	0.713	0.942	0.544	0.726	77
OURS	0.741	0.746	0.942	0.582	0.753	48

**Table 2:** Ablation study on our dataset.

Method	Linear	Dotted	Sludge	Pinhole	mAP	FPS	GFLOPs	Params (M)
YOLOv5s (baseline)	0.704	0.713	0.942	0.544	0.726	77	15.8	7.0
+C3 ECA	0.729	0.712	0.939	0.562	0.735	71	15.8	7.0
+GSConv+SlimNeck	0.715	0.732	0.945	0.587	0.745	67	14.5	6.8
+ASFF Detect	0.741	0.746	0.942	0.582	0.753	48	22.9	12.2

## Data Availability

The data used to support the findings of this study are available from the corresponding author upon reasonable request (lfc@ysu.edu.cn).

## References

[1] Sun X., Gu J., Huang R., Zou R., Giron Palomares B. (2019). Surface defects recognition of wheel hub based on improved faster r-cnn. Electronics.

[2] Jin C., Kong X., Chang J., Cheng H., Liu X. (2020). Internal crack detection of castings: A study based on relief algorithm and adaboost-svm. Int. J. Adv. Manuf. Technol..

[3] Yun L., Yan B., Dan Q., Liu F. Research on fault diagnosis of photovoltaic array based on random forest algorithm. Proceedings of the IEEE International Conference on Power Electronics, Computer Applications (ICPECA).

[4] Liu W., Anguelov D., Erhan D., Szegedy C., Reed S., Fu C.-Y., Berg A.C., Leibe B., Matas J., Sebe N., Welling M. (2016). SSD: Single shot multibox detector. Computer Vision–ECCV 2016: 14th European Conference, Amsterdam, The Netherlands, 11–14 October 2016.

[5] Redmon J., Divvala S., Girshick R., Farhadi A. You only look once: Unified, real-time object detection. Proceedings of the IEEE Conference on Computer Vision and Pattern Recognition.

[6] Redmon J., Farhadi A. YOLO9000: Better, faster, stronger. Proceedings of the IEEE Conference on Computer Vision and Pattern Recognition.

[7] Redmon J., Farhadi A. (2018). YOLOv3: An incremental improvement. arXiv.

[8] Bochkovskiy A., Wang C.-Y., Liao H.-Y.M. (2020). YOLOv4: Optimal speed and accuracy of object detection. arXiv.

[9] Girshick R., Donahue J., Darrell T., Malik J. Rich feature hierarchies for accurate object detection and semantic segmentation. Proceedings of the IEEE Conference on Computer Vision and Pattern Recognition.

[10] Girshick R. Fast R-CNN. Proceedings of the IEEE International Conference on Computer Vision.

[11] Ren S., He K., Girshick R., Sun J. (2015). Faster R-CNN: Towards real-time object detection with region proposal networks. Advances in Neural Information Processing Systems.

[12] Ding F., Zhuang Z., Liu Y., Jiang D., Yan X., Wang Z. (2020). Detecting defects on solid wood panels based on an improved SSD algorithm. Sensors.

[13] Li Y., Wang Z. (2021). Research on textile defect detection based on improved cascade R-CNN. Proceedings of the International Conference on Artificial Intelligence and Electromechanical Automation (AIEA), Zhangjiajie, China, 28–30 May 2021.

[14] Fan S., Liang X., Huang W., Zhang V.J., Pang Q., He X., Li L., Zhang C. (2022). Real-time defects detection for apple sorting using NIR cameras with pruning-based YOLOv4 network. Comput. Electron. Agric..

[15] Guo Z., Wang C., Yang G., Huang Z., Li G. (2022). MSFT-YOLO: Improved YOLOv5 based on transformer for detecting defects of steel surface. Sensors.

[16] Cheng X., Yu J. (2021). Retinanet with difference channel attention and adaptively spatial feature fusion for steel surface defect detection. IEEE Trans. Instrum. Meas..

[17] Liu M., Li Z., Li Y., Liu Y. (2022). A fast and accurate method of power line intelligent inspection based on edge computing. IEEE Trans. Instrum. Meas..

[18] Hu J., Shen L., Sun G. Squeeze-and-excitation networks. Proceedings of the IEEE Conference on Computer Vision and Pattern Recognition.

[19] Woo S., Park J., Lee J.-Y., Kweon I.S. CBAM: Convolutional block attention module. Proceedings of the European Conference on Computer Vision (ECCV).

[20] Wang Q., Wu B., Zhu P., Li P., Zuo W., Hu Q. ECA-Net: Efficient channel attention for deep convolutional neural networks. Proceedings of the IEEE/CVF Conference on Computer Vision and Pattern Recognition.

[21] Li H., Li J., Wei H., Liu Z., Zhan Z., Ren Q. (2022). Slim-neck by GSConv: A better design paradigm of detector architectures for autonomous vehicles. arXiv.

[22] Liu S., Huang D., Wang Y. (2019). Learning spatial fusion for single-shot object detection. arXiv.

[23] Everingham M., Van Gool L., Williams C.K.I., Winn J., Zisserman A. (2010). The Pascal Visual Object Classes (VOC) Challenge. Int. J. Comput. Vis..

